# In-Depth Analysis of Cement-Based Material Incorporating Metakaolin Using Individual and Ensemble Machine Learning Approaches

**DOI:** 10.3390/ma15217764

**Published:** 2022-11-03

**Authors:** Abdulrahman Mohamad Radwan Bulbul, Kaffayatullah Khan, Afnan Nafees, Muhammad Nasir Amin, Waqas Ahmad, Muhammad Usman, Sohaib Nazar, Abdullah Mohammad Abu Arab

**Affiliations:** 1 Department of Civil and Environmental Engineering, College of Engineering, King Faisal University, Al-Ahsa 31982, Saudi Arabia; 2 Department of Civil Engineering, COMSATS University Islamabad, Abbottabad 22060, Pakistan; 3 Interdisciplinary Research Center for Hydrogen and Energy Storage (IRC-HES), King Fahd University of Petroleum & Minerals (KFUPM), Dhahran 31261, Saudi Arabia

**Keywords:** metakaolin, SHAP analysis, bagging, boosting, decision tree, multilayer perceptron neural network, random forest

## Abstract

In recent decades, a variety of organizational sectors have demanded and researched green structural materials. Concrete is the most extensively used manmade material. Given the adverse environmental effect of cement manufacturing, research has focused on minimizing environmental impact and cement-based product costs. Metakaolin (MK) as an additive or partial cement replacement is a key subject of concrete research. Developing predictive machine learning (ML) models is crucial as environmental challenges rise. Since cement-based materials have few ML approaches, it is important to develop strategies to enhance their mechanical properties. This article analyses ML techniques for forecasting MK concrete compressive strength (fc’). Three different individual and ensemble ML predictive models are presented in detail, namely decision tree (DT), multilayer perceptron neural network (MLPNN), and random forest (RF), along with the most effective factors, allowing for efficient investigation and prediction of the fc’ of MK concrete. The authors used a database of MK concrete mechanical features for model generalization, a key aspect of any prediction or simulation effort. The database includes 551 data points with relevant model parameters for computing MK concrete’s fc’. The database contains cement, metakaolin, coarse and fine aggregate, water, silica fume, superplasticizer, and age, which affect concrete’s fc’ but were seldom considered critical input characteristics in the past. Finally, the performance of the models is assessed to pick and deploy the best predicted model for MK concrete mechanical characteristics. K-fold cross validation was employed to avoid overfitting issues of the models. Additionally, ML approaches were utilized to combine SHapley Additive exPlanations (SHAP) data to better understand the MK mix design non-linear behaviour and how each input parameter’s weighting influences the total contribution. Results depict that DT AdaBoost and modified bagging are the best ML algorithms for predicting MK concrete fc’ with R^2^ = 0.92. Moreover, according to SHAP analysis, age impacts MK concrete fc’ the most, followed by coarse aggregate and superplasticizer. Silica fume affects MK concrete’s fc’ least. ML algorithms estimate MK concrete’s mechanical characteristics to promote sustainability.

## 1. Introduction

Throughout the previous decades, there has been a strong demand and concern for investigation to develop green structural materials to meet the increasing need from public and private sectors. Concrete continues to be the most widely utilized manmade substance on the planet. Given the considerable environmental impact of cement production, research has concentrated on both reducing the impact on the environment and cost reductions for cement-based products [1,2,3]. The utilization of metakaolin (MK) as an additive or partial substitute for cement is a major area of research in the manufacture of concrete materials.

**Figure 1:**
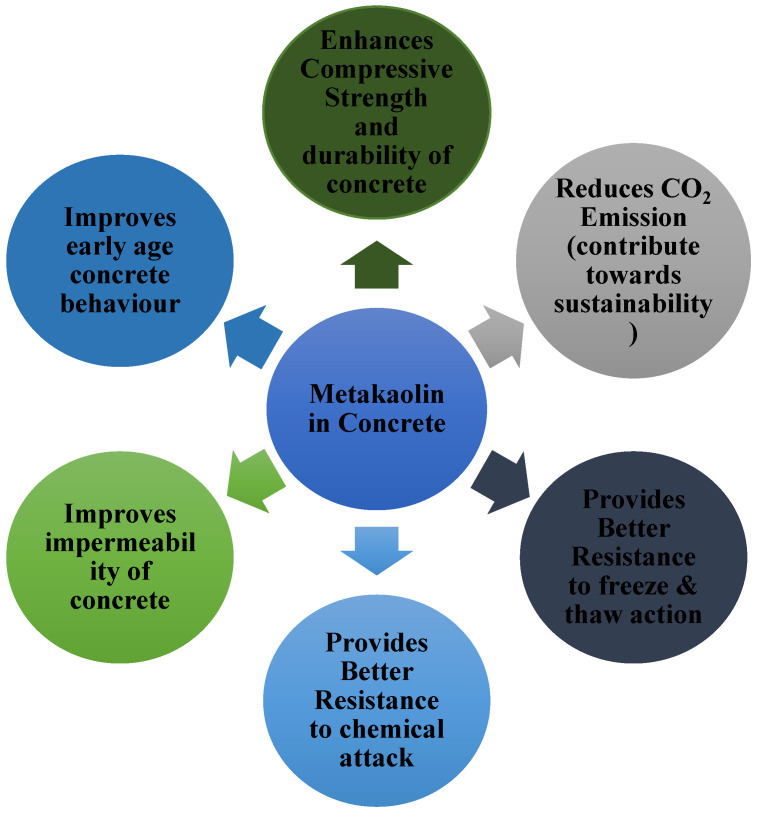
Advantages of metakaolin in concrete.

MK is an alternative to cement that is manufactured by calcining kaolin clays at elevated temperatures ranging from 700 °C to 900 °C. As a cement replacement in concrete structures, MK has been employed as a 10% to 50% replacement, depending on the specific application [4,5,6,7]. It has been shown that MK enhances the mechanical and durability properties when used in place of Portland cement [8,9,10]. The pozzolanic reaction, MK aggregate’s fineness, and the accelerated cement hydration all contribute to an increase in concrete’s compressive strength (fc’) during the early curing phases [11]. Additionally, cement manufacture generates a substantial amount of carbon dioxide (CO_2_) emissions; this new trend of replacing metakaolin for cement in concrete is part of a comprehensive approach to environmental sustainability. Addition of MK in concrete has various advantages as depicted in Figure 1.

The cost, labour, and time consuming complexity of laboratory-based mixture optimisation might be replaced by computational modelling techniques [12]. To determine the optimum concrete mixtures, these approaches generate objective functions from the concrete components and their properties, and then use optimization techniques to determine the best concrete mixtures. Previously, goal functions for linear and nonlinear models were individually created. Due to the very nonlinear connections between concrete qualities and input parameters, the relationships of such models cannot be precisely established. Therefore, researchers are using machine learning (ML) techniques for predicting concrete properties.

Creating a concrete mix with MK in it complicates the determination of the concrete’s fc’ using an analytical formula, as opposed to a standard concrete, which has fewer mix parameters than cement MK specimen. This is mostly because of the enormous number of constituents and the fc’ very nonlinear behaviour in regard to the mix parameters. To this purpose, when basic equations cannot directly connect the input and output values, machine learning (ML) techniques frequently give important alternatives in the context of engineering problem solving [13,14,15,16,17,18,19,20,21,22]. Owing to the intricate nonlinear interactions amongst independent and dependent variables, such techniques can be accomplished with a sufficient level of accuracy if a comprehensive library of sufficient experimental data points is accessible in the area of computational engineering structures and materials. Thus, a wide range of innovative approaches to a wide range of technological problems may be put into practice.

Until now, the literature has primarily focused on the use of ML techniques such as artificial neural network (ANN) in the field of materials science without ensemble learners [23,24,25,26]. These algorithms were utilized to predict the fc’ and elasticity modulus of materials composed of cement [26,27,28,29]. The literature has comprehensive and extensive publications on the use of ANNs in the modelling of concrete materials [30,31,32,33,34,35]. Fuzzy logic algorithms and genetic algorithms approaches have also been utilized in the recent decade in place of ANN models to describe the mechanical properties of cement-based materials [36,37,38,39,40,41].

Since cement-based materials have a limited number of ML methods, it is vital to investigate if other ML techniques may be used to improve their mechanical characteristics. Thus, the present work investigates ML approaches application for predicting the fc’ of MK concrete. Three different individual and ensemble ML predictive models are presented in detail, namely decision tree (DT), multilayer perceptron neural network (MLPNN), and random forest (RF), together with the factors that are most effective, allowing for efficient investigation and prediction of the fc’ of cement-based concrete. The authors employed a comprehensive database of MK concrete mechanical characteristics for model generalisation since it is an essential part of any prediction or simulation work. The reported database contains 551 data points with highly effective input parameters for calculating the fc’ of MK concrete. The database includes a value for cement, metakaolin, coarse and fine aggregate, water, silica fume, superplasticizer, and age, which have a considerable effect on the fc’ of concrete and have rarely been treated as vital input parameters in the past. The trained and created model has produced a holistic map of concrete fc’. Finally, the performance capabilities of the offered models are evaluated in order to select and implement the most predictive model for addressing the mechanical properties of MK concrete.

There has been a surge in increased interest in large-scale production of sustainable, low-priced, and high-performance construction materials that are also robust in adverse ecological circumstances over the previous few decades. One of the world’s most common construction materials—cement-based concrete—required the incorporation of more components and additives than previously used concrete because of environmental concerns. However, the high number of mixture factors and their substantially nonlinear relationship to the mechanical characteristics of concrete, such as the fc’, challenge the analytical methods for numerically estimating the concrete fc’. To this purpose, unconventional methods become a critical instrument for resolving the afore-mentioned complicated optimisation problem. In this perspective, the most widely used ML techniques, such as, DT, MLPNN, and RF, have been suggested for estimating the fc’ of concrete, a critical parameter for the reliable design in structure. Among the proposed ML models, the optimal predictive model has shown to be extremely successful, demonstrating trustworthy projections and, most importantly, showing its highly non-linear mechanical properties.

Additionally, there seems to be a research gap in the study of MK fc’ and its influence on raw materials. It was, thus, necessary to investigate the influence of MK containing concrete’s input parameters/raw components on its anticipated compressive strength using a post hoc model-agnostic approach known as SHapley Additive exPlanations (SHAP) [42,43]. Machine learning (ML) techniques were used to integrate SHAP data in order to get a better understanding of the multifarious non-linear behaviour of the MK design mix for the strength parameter and how each input parameter’s weighting affects the overall contribution. ML approaches may be used to accurately forecast concrete kinds, as previously stated. The experimental setting requires a significant investment in terms of labour, time, and resources to do this. Data modelling and the discovery of interconnected independent components, as well as a rapid reduction in input matrix size are, thus, urgently required. Concrete construction materials may be accurately predicted using machine learning approaches. The use of ML methods may be justified as an alternate strategy to calculating MK fc’ in order to save on both time and money spent on experiments. We used both a stand-alone ML model and an ensemble of ML models in our investigation. Additionally, statistical tests were used to evaluate the models, and their results were compared. Later, a model with precise MK prediction was suggested based on the performance of several statistical factors. In order to get a thorough understanding of mix design in order to achieve MK concrete strength, this research also explained how input factors contributed and how ML models were integrated. Explainable ML techniques and features significance for considerable characteristics of the structure were found to be linked in the study’s overall findings.

## 2. Data Description

**Figure 2:**
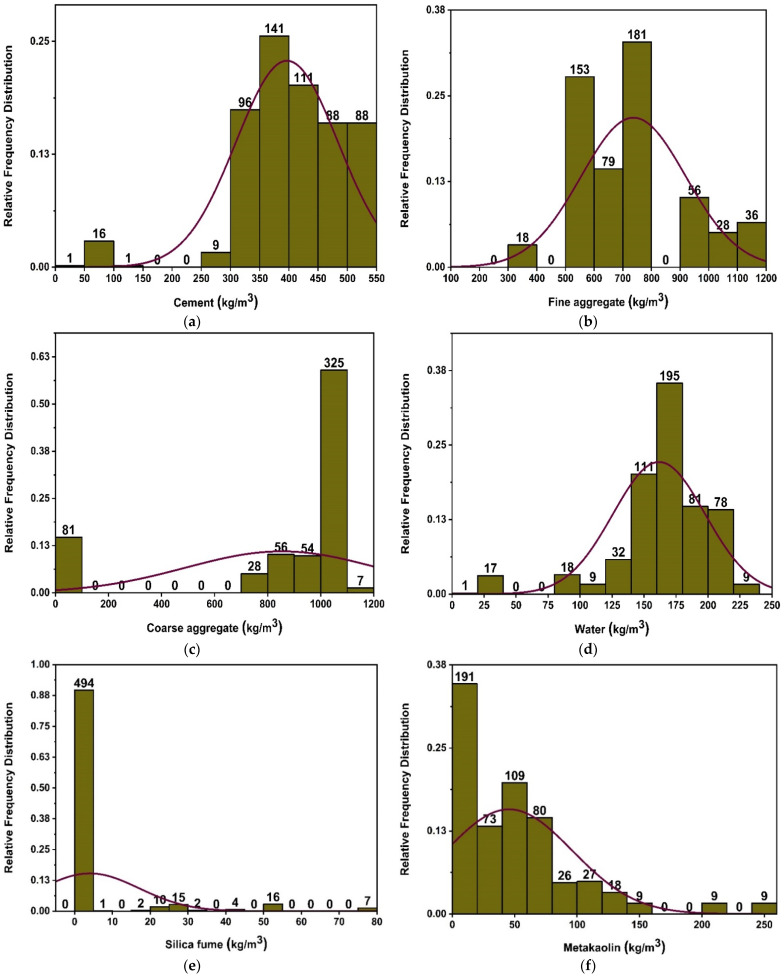

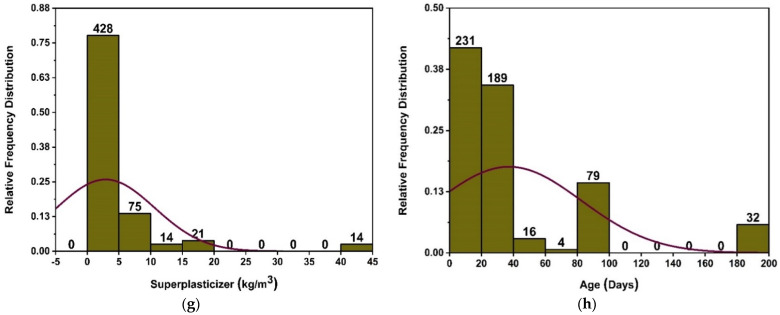
Compressive strength parameters’ relative frequency distribution; (**a**) cement, (**b**) fine aggregate, (**c**) coarse aggregate, (**d**) water, (**e**) silica fume, (**f**) metakaolin, (**g**) superplasticizer, and (**h**) age.

Currently accessible literature has been used to get the data needed to simulate concrete’s fc’ utilising MK [44,45,46,47,48,49,50,51,52,53,54,55]. The predicted output compressive strength data consists of eight input parameters, which include cement, MK, fine and coarse aggregate, water, age of concrete, superplasticizer, and silica fume. Type of cement is not considered as an input parameter, as only one type of cement (Type-I) is utilized for modelling the ML algorithms. Cement and metakaolin are two constituents that are prevalent among those selected for the database. Additionally, attempts were made to choose articles that share common components (admixtures, superplasticizers, etc.). The authors tried to choose publications based on criteria related to materials that are widely used in concrete and make important contributions to concrete’s mechanical characteristics. Further, similar material in varied arrangements is required for modelling ML algorithms. Except for age in days, all characteristics are measured in kilograms/m^3^. Descriptive statistics are a set of descriptive coefficients that provide a result that may be applied to the whole population or to a sample of the population. In descriptive statistics, measures of central tendency and measures of variability are used (spread). However, variance, standard deviation, maximum and minimum variables, kurtosis, and skewness are all indices of variability. Table 1 and Table 2 and Figure 2 provide the variation in data used to run the models. Various information is reflected in the descriptive analysis’s outcomes, which are derived from the data of all the input variables. Additionally, the table displays the ranges, maximum, and lowest values of each model variable. Nonetheless, the other parameters of the study, such as mean, mode, standard deviation, and the total of all data points for each variable, also reveal the important values. Figure 2 depicts the relative frequency distribution of each parameter utilised in the mixes. A relative frequency distribution illustrates the percentage of total observations that correspond to each value or class of values. It has tight ties to a probability distribution, which is often used in statistics. Figure 2 depicts the link of input parameters by displaying the relative frequency distribution of data items. Each chosen parameter has a significant impact on the concrete’s strength characteristics. In addition, Table 1 displays the lowest and highest variable values including 551 datasets, and Table 2 provides a data analysis check with the variance, range, standard deviation, and mean.

## 3. Methodology

**Figure 3:**
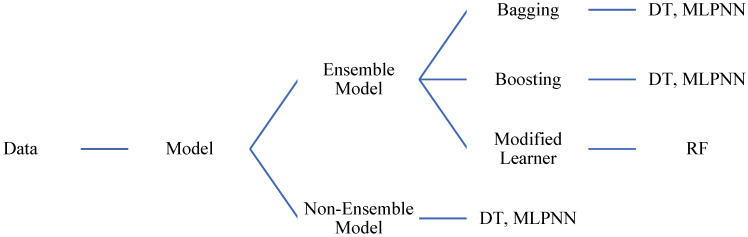
Flow chart of ML techniques.

ML techniques are being used in a variety of fields to anticipate and understand the behaviour of materials. In this work, ML-based techniques comprising of the DT, MLPNN, and RF are employed for forecasting the fc’ of MK concrete. The selection of these methods is based on their prevalence and reliability in the forecast of outcomes in comparable studies, as well as their significance as the top data mining algorithms. In addition, an ensemble algorithm was afterwards employed to simulate the concrete fc’. Figure 3 displays the technique flow chat for ensemble learning.

### 3.1. Machine Learning Methods

It has been shown that artificial intelligence (AI) is a more effective modelling methodology than traditional methods. AI has a number of advantages for addressing ambiguous difficulties and is an excellent method for handling such complicated situations. It is possible to identify engineering design parameters using AI-based methods when testing is not possible, resulting in significantly reducing the workload of human testers. In addition, AI could expedite decision making, decrease error, and improve processing efficacy [56]. Recently, a rise of interest in the application of artificial intelligence to all scientific fields has been observed, sparking a range of goals and aspirations. The field of civil engineering has experienced a significant increase for utilizing different AI methods all over its numerous fields. ML, reinforcement learning, and deep learning (DL) are three AI approaches that are proving to be a new category of creative approaches to structural engineering problems. ML is a fast-expanding area of AI that is often used in the construction sector for predicting behaviour of material. One project aims to investigate inclusion of social elements into multi criteria infrastructure assessment strategies, with inclusion of social factors into the assessment of infrastructure’s long-term viability using multi-criteria assessment techniques [57]. In the framework of structural design, exhaustive study on evolutionary computation, an area of artificial intelligence, was conducted [58]. Yin et al. [59] explored AI uses in geotechnical engineering. A study was done to determine the state of high-rise building optimization. [60]. In order to synthesise concepts in the developing field of AI applications in civil engineering, this study was done. This list contains a broad variety of methods: Fuzzy systems (FS), neural networks, expert systems (ES), reasoning, categorization, and learning are only a few examples of evolutionary computing [61].

In spite of the fact that the referenced review papers discussed the use of AI in civil engineering, they mostly concentrated on the usage of old approaches and did not cover latest methods using ensemble techniques. Figure 3 shows an estimation of the fc’ of MK concrete using ML approaches including DT, MLPNN, and RF. These algorithms were selected on the basis of their broad usage in relevant research and their reputation as the finest data prediction algorithms available. In addition, ensemble techniques are employed to predict the concrete modelling strength. In terms of computational speed and processing time, ML models are fairly significant. Compared to conventional models, the rate of error is almost non-existent. A comparison is made among individual and ensemble models in this research. SHAP analysis is performed to find the optimum dosage and contribution of each input parameter towards fc’. Moreover, positive and negative impacts of each input parameter and their effect on other parameters are also studied.

#### 3.1.1. Decision Tree-Based Machine Learning

In DT, any number of nodes can be connected to any number of branches, and each node can have an infinite number of branches. Leaves are the nodes that do not have any outgoing ends, whereas inner nodes are those that do. Using an interior node for a specific event, the case utilized for classification or regression can be partitioned into multiple classes. During the learning process, the input variables play an important role. The algorithm that generates the DT from provided instances serves as the stimulant for the DT. By reducing the fitness function, the implemented algorithm calculates the optimal DT. A regression model is used because there are no classes in the dataset selected for this study, so the independent variables are used instead of the target variable. For each variable, the dataset is broken up into many subsets. The error among the anticipated and the actual values of the pre-specified relation is determined at every split point by the algorithm. The variable having least values of fitness function is selected as the split point after comparing the inaccuracies in the split point across the variable quantity. Repeatedly, this technique is carried out.

In the DT architecture, independent variables are partitioned into homogenous zones by decision rules that recursively split them [62]. DT is primarily concerned with the investigation of a system for making decisions that are suitable for predicting a result given a collection of inputs. DT is referred to as a regression tree or a classification tree, depending on whether the target variables are continuous or discrete [63]. Numerous studies have demonstrated the effectiveness of DT in a variety of real-world situations for the aim of prediction and/or categorization [64].

The primary advantage of DT is its ability to simulate complex interactions between existent variables. Through consideration of how data are distributed, DT models are capable of combining both continuous and categorical variables without making any stringent assumptions [65]. Additionally, developing a DT is straightforward, and the resulting models are easily interpretable. Additionally, when it comes to determining the relative relevance of input characteristics, DT is an excellent choice [66].

**Figure 4:**
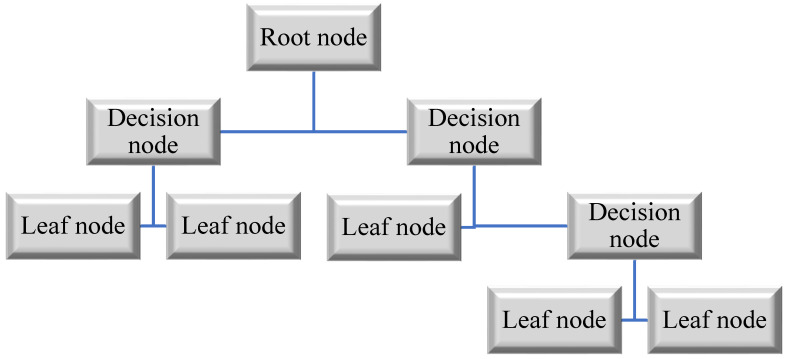
Flow chart of DT.

DT modelling includes two phases: tree creation and tree pruning [67]. Stage one starts with DT’s root node being identified as the independent variables with the highest performance gain. Following that, according to root values, the training dataset is partitioned into subsets and sub-nodes are created. When the input variables are discrete, a sub-node of the tree is constructed for each conceivable value, whereas in some circumstances, the threshold-finding step results in the generation of two sub-nodes [68]. Following that, each sub-gain node’s share is calculated, and the technique is recurring till all samples in a certain node are classified as belonging to the same class. They are then referred to as “leaf nodes,” and their values are designated as the values of the classes they belong to. A flow chart of DT is shown in Figure 4.

#### 3.1.2. Artificial Neural Network-Based Machine Learning

**Figure 5:**
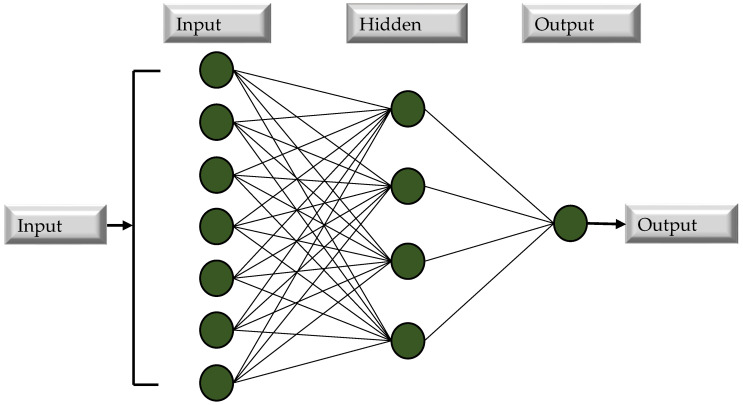
Flow chart of MLPNN.

A multilayer perceptron neural network (MLPNN), a network-based DM computing approach, is employed in this research with individual base learner and ensemble base learner methods to model and forecast MK concrete. An ANN program mimics the structure of a biological nervous system’s neurons [69]. Parallel linkages provide the basis of ANNs. In order to transmit the weighted inputs from neurons, these cells use an activation function to transmit the weighted outputs. It is possible to have one or more multi-layers in these activities. Use of the multilayer perceptron network is widespread in brain activity. The perceptual response is created among the number of input parameters and the number of output parameters. There are three types of layers in a network: input, hidden, and output. Between the input and output layers, there is a hidden layer that may have a huge network of hidden layers. The perceptron can handle all of its issues with a single layer, but it is more efficient and helpful to have several hidden layers [70]. Figure 5 depicts a typical neural network design. With the exception of neurons in the input layer, all neurons in a layer perform linear addition and bias computations. Non-linear functions are then calculated in the hidden layers by neutrons A sigmoid function is a word used to describe a non-linear function [71]. This research paper models ANNs using a feed-forward multi-layer perceptron (MLPNN) network. To discover the highest-performing MLPNN, hidden layers and neuron pairs of varied numbers are meticulously selected [72]. In the hidden layer, the link between input and output variables may also be determined using a linear activation function and a nonlinear transit function. In addition, the data extracted from the published literature are separated into training and testing sets. This is conducted to reduce the influence of data overfitting, since overfitting is an intractable issue in machine learning. Randomly, 80 percent of the data is used for training the models and 20 percent for testing the trained models, as recommended by the literature [73].

### 3.2. Bagging and Boosting for Ensemble Approaches

ML classification and prediction accuracy may be improved using ensemble approaches. By combining and accumulating several weaker predictive models, such techniques frequently support decrease training data over-fitting problems (component sub-models). Training data may be manipulated to create various sub-model/classifier components (i.e., 1 to M) that help a learner. Furthermore, the best predictive model may be generated by merging limiting sub-models using averaging combination processes. Bootstrap resampling and benefit collection are two common methods for modelling ensembles that make use of bagging. The first training set substitute’s component replicates the bagging procedure. In product models, specific data samples might appear numerous times, while others do not. An average is computed from the output of each component model. As with the bagging method, this strategy builds a cumulative model that yields several components with more accuracy as compared to the individual model. A weighted average of dependent sub-models is used to set sub-models in the final model, which is referred to as “boosting”. AdaBoost [74] is a rapid ensemble learning algorithm that picks multiple classifier examples repeatedly by distributing weights adaptively across training cases. This approach linearly combines the chosen classifier instances to form an ensemble. Even when a large number of base classifier instances are included in a model, AdaBoost ensembles seldom display an overfitting issue [75]. It is possible to diminish loss function by fitting to a staged additive model. This indicates that a cost function that is not differentiable and is not smoothed has been optimized; it can best be described using an exponential loss function [75]. It is, therefore, possible to employ AdaBoost to tackle a number of classification problems with impressive results. DT, MLPNN, and RF are used in conjunction with ensemble learners to envisage the strength of consistently used concrete in this study.

### 3.3. Ensemble Learner’s Parameter Tuning

**Figure 6:**
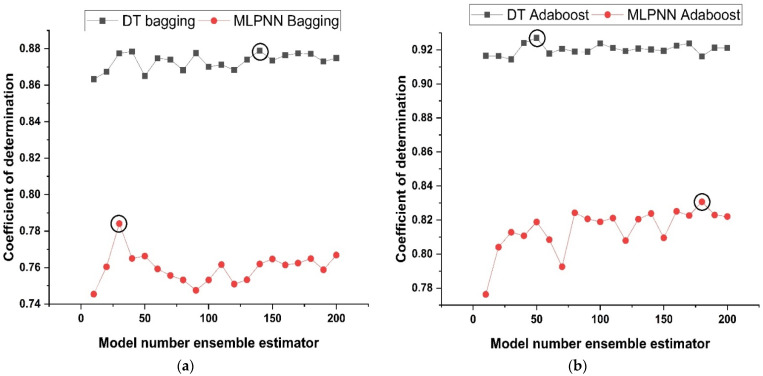
ML with ensemble sub-models; (**a**) Bagging; (**b**) AdaBoost.

Models of the tuning parameters that are employed in the ensemble methods might consist of (i) factors connected with the optimum model learners’ number and (ii) rate of learning and other attributes that have a significant influence on ensemble algorithms. In this research, twenty sub-models were generated for each ensemble base learner. The component sub-models ranged in size from 10 to 200, and the optimum constructs were selected on the basis of the large values of determination coefficient. Figure 6 illustrates the link between performance of ensemble model and the number of component sub-models. As shown in Figure 6a,b, the assembling model with bagging and boosting yields a significant determination coefficient in the estimation terms. As demonstrated in Figure 6a, the 140th sub-model of DT with bagging as an ensemble of other sub-models provides a stronger relationship than the other sub-models of DT with bagging. Similarly, the 30th sub-model of MLPNN with bagging provides a significant higher correlation coefficient as compared to MLPNN bagging other sub-models. Similarly, as depicted in Figure 6b, the 50th DT AdaBoost and the 180th MLPNN AdaBoost sub-model provide the best results when compared to their other sub-models. Preliminary analysis indicates that the usage of ensemble modelling improves the efficiency of both models.

### 3.4. Random Forest Regression Based Machine Learning

The RF model is a regression and classification strategy that has piqued the curiosity of a number of different researchers [76]. The main difference between DT and RF is that one tree is created in DT, but several trees are built in RF, and unlike data are randomly picked and distributed to all the trees in the forest. Each tree’s data are organised into columns and rows, with a variety of column and row sizes to choose from [77]. Each stage of a tree’s development is detailed below:

1. The equivalent of two-thirds of the entire dataset is chosen for each tree at random. Bagging is the term used to describe this practise. Predictor variables are picked, and node splitting is done based on the best possible node split on these variables.
2. The remaining data are used to estimate the out-of-bag error for each and every tree. To get the most accurate estimation of the out-of-bag error rate, errors from each tree are then added together.
3. Every tree in the RF algorithm provides a regression, but the model prioritizes the forest that receives the most votes over all of the individual trees in the forest. The votes might be either zeros or ones. As a prediction probability, the fraction of 1s achieved is provided.

### 3.5. 10 K Fold Method for Cross Validation

**Figure 7:**
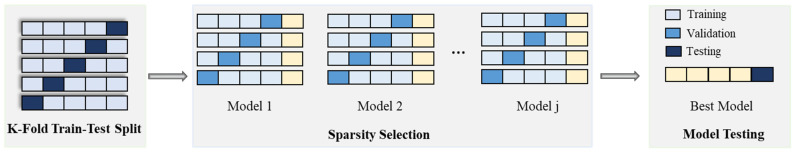
K-fold cross validation method [78].

For training and holdout data, the k fold approach for cross validation is often employed to decrease arbitrary sampling prejudice. A stratified 10-fold cross-validation strategy was used in this work to evaluate model performance by dividing the input data into ten distinct subsets. Each of the 10 rounds of model construction and validation uses a different sample of data to test and train the model. As indicated in Figure 7, in order to validate the adequacy of the model, the test subset is utilised. The accuracy of algorithm is calculated as the mean of the 10 models’ accuracy scores after 10 rounds of validation.

### 3.6. Evaluation Criteria for Models

Statistical errors for example root mean squared logarithmic error (RMSLE), square value (R^2^), mean absolute error root (MAE), and root mean square error (RMSE) are used to assess model performance on a training or testing set. R^2^ is also called the determination coefficient and is used to evaluate a model’s ability to predict. Concrete’s mechanical characteristics may now be predicted with greater accuracy due to advances in artificial intelligence modelling methods. The models are assessed statistically by calculating error metrics in this research. There are a variety of measures that might help us better understand the model’s inaccuracy. In addition, the model’s performance may be assessed using the variance coefficient and standard deviation. According to the coefficient of determination, the model’s correctness and validity may be confirmed. Models with R^2^ values ranging from 0.65 to 0.75 indicate promising results, whereas models with R^2^ values lower than 0.50 reveal disappointing outcomes. Equation (1) can be used to determine R^2^. The units used in MAE are the same as the ones used in the output. It is possible for a model with a value of MAE that falls within a certain limit to have large errors at some points in time. In order to calculate MAE, Equation (2) is used. RMSE is the under root of the average of squared differences between estimates and measurements. Error squared is calculated by summing the squared errors. This method gives a greater weight to outliers and significant exceptions than other methods, which results in bigger squared differences in certain cases and lower squared differences in others. Using RMSE, the model’s average estimation error given an input can be calculated. Improved models have fewer root mean squared errors of variation. The lower the value of RMSE, the less accurate the model is in predicting the data. Equation (3) is used to determine RMSE. Relative imprecision amongst forecasted and actual values is taken into account by RMSLE. It is the difference between the expected value and the actual value, expressed as a logarithmic scale. RMSLE is calculated using Equation (4).

(1) $$R^{2}=\frac{\sum_{i=1}^{n}\left(M_{i}-\;{\bar{M}}_{i}\right)(P_{i}-\;{\bar{P}}_{i})}{\sqrt{\sum_{i=1}^{n}{\left(M_{i}-\;{\bar{M}}_{i}\right)}^{2}\sum_{i=1}^{n}{\left(P_{i}-\;{\bar{P}}_{i}\right)}^{2}}}$$

(2) $$\mathrm{MAE}=\frac{1}{n}\sum_{i=1}^{n}\left|P_{i}-M_{i}\right|\;$$

(3) $$\mathrm{RMSE}=\sqrt[{2}]{\frac{\sum_{i=1}^{n}{\left(P_{i}-M_{i}\right)}^{2}}{N}}$$

(4) $$\mathrm{RMSLE}\;=\sqrt{\frac{1}{N}\overset{N}{\underset{i=1}{\sum }}{\left(\log\left(\mathrm{yi}+1\right)-\log\left(\mathrm{yj}+1\right)\right)}^{2}}\;$$

## 4. Model Result

### 4.1. Decision Tree Model Outcomes

**Figure 8:**
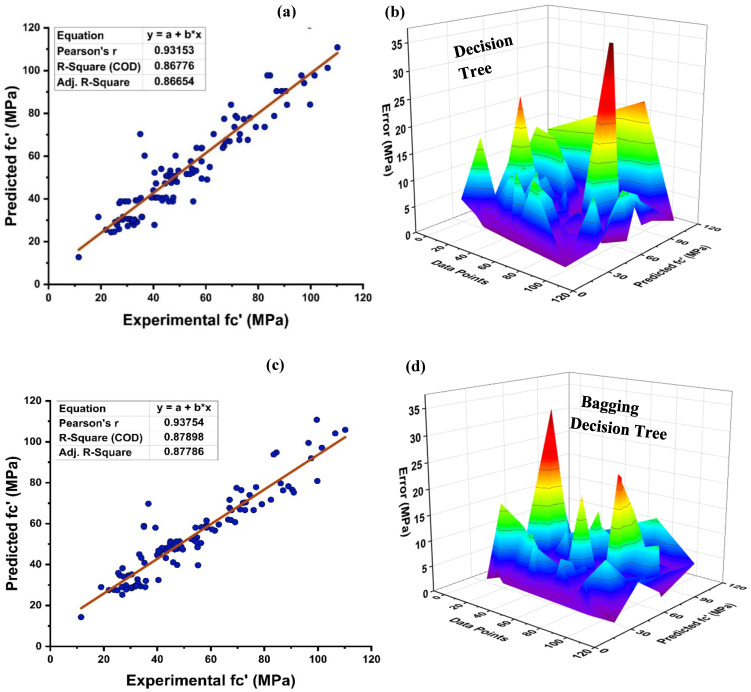

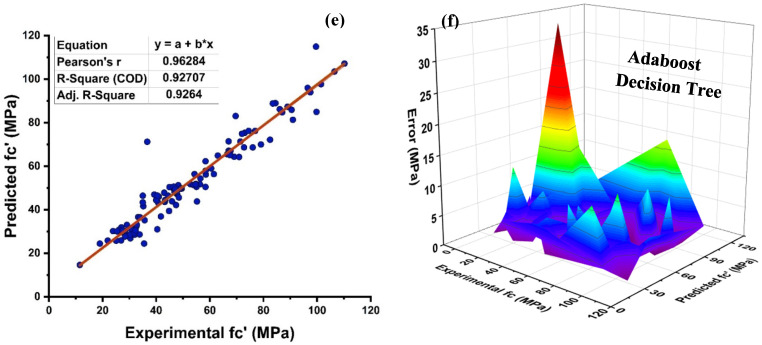
(**a**) DT individual base learner regression model; (**b**) DT individual base learner regression model error distribution; (**c**) DT-bagging model; (**d**) DT-bagging model error distribution; (**e**) DT-AdaBoost regression model; and (**f**) DT-AdaBoost model error distribution.

As seen in Figure 8, the DT is modelled using various ensemble techniques including bagging and boosting. The actual prediction from individual base learner DT produces a high relationship with predicted values having a R^2^ = 0.868, as seen in Figure 8a. Figure 8b depicts the error distribution of an individual DT model. Figure 8b indicates that the testing set has an average inaccuracy value of 5.79 MPa. In addition, 82.88 percent of the data exhibit error below 10 MPa, and 11.7% of the data exhibit error between 10–15 MPa. In contrast, each domain of 15–20 MPa, 20–25 MPa, and 35–40 MPa contains 1.8 percent data error, with a maximum and minimum error of 35.3 MPa and 0.085 MPa, respectively, as illustrated in Figure 8b. Individually, DT provide accurate predictions; but, if the DT is an ensemble of several methodologies, it yields a more precise outcome, as seen in Figure 8c–f. Bagging ensemble yields a conclusive and favourable result with R^2^ = 0.879 and minimal testing data error. The data indicate an inaccuracy of 84.685% below 10 MPa, 9% between 10 and 15 MPa, and 3.6% between 15 and20 MPa. As shown in Figure 8d, only 1.8% of the data fall between 20 and 25 MPa and 0.9% between 30 and 35 MPa, with a maximum and minimum error of about 33.06 MPa 0.029 MPa, respectively. Similar to individual DT and bagging DT algorithms, boosting with AdaBoost produces models with a significant correlation. As seen in Figure 8e–f, this is because of the influence that a strong learner has on the aspect of prediction. A DT AdaBoost ensemble model has a R^2^ equal to 0.924. The error distribution is minimised by applying AdaBoost with a DT, with an average error of 4.12 MPa, a maximum and minimum error of 34.578 MPa, and 0.065 MPa, respectively. Approximately 92.79%of the data is below 10 MPa, with 6.3% between 10 and 15 MPa and 0.9% between 30 and 35 MPa. Table 3 presents the statistical information pertaining to DT with bagging and boosting ensemble learners.

### 4.2. MLPNN Model Outcomes

**Figure 9:**
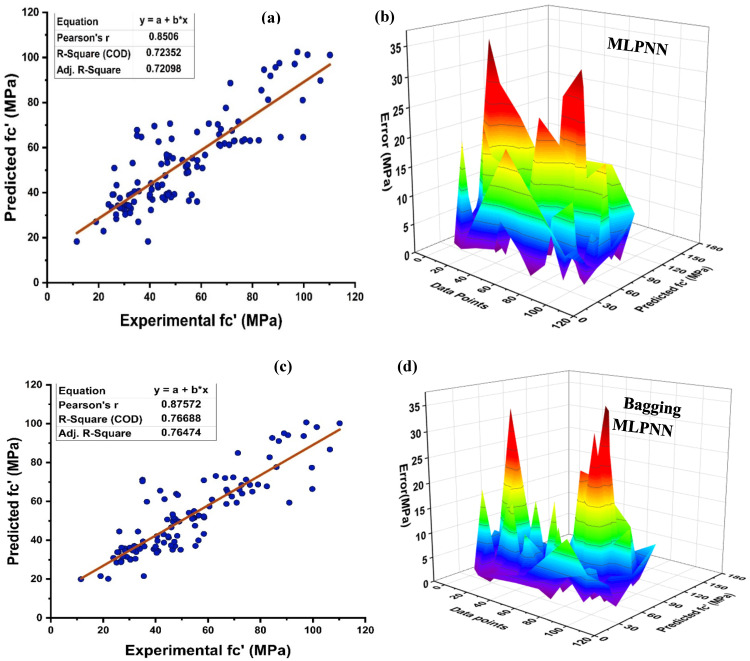

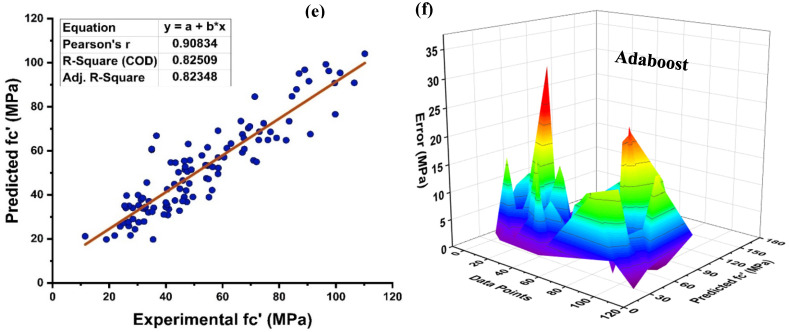
(**a**) MLPNN individual base learner regression model; (**b**) MLPNN individual base learner regression model error distribution; (**c**) MLPNN-bagging regression; (**d**) MLPNN-bagging regression model error distribution; (**e**) MLPNN-AdaBoost regression model; (**f**) MLPNN-AdaBoost regression model error distribution.

In the field of ML and AI, neural networks fall under the rubric of supervised learning, and its implementation yields a rigid correlation between prediction and target response. As illustrated in Figure 9, MLPNN is also modelled utilising ensemble learner’s methods, similar to the DT. Figure 9a depicts the actual projection of MK concrete with R^2^ = 0.724 with its error distribution as seen in Figure 9b. MLPNN error distribution indicates that a test set has an average error of 8.70 MPa, with lowest and highest errors of 0.044 MPa and 35.15 MPa, respectively. However, MLPNN ensemble model reduces the distribution of average error with a rise in the R^2^ of around 0.767 for bagging and 0.825 for boosting, respectively. The average error for MLPNN-bagging and AdaBoost boosting is 7.29 MPa and 7.05 MPa, respectively, as seen in Figure 9c–f. In addition, a major portion of testing set error is below 10 MPa, with 72.97%, 77.48%, and 74.77% of the data, respectively, for the individual, bagging, and AdaBoost MLPNN models. These ensemble-model outputs also demonstrate a rise in R^2^ by exhibiting less inaccuracy than the real output. Table 4 illustrates the statistical evaluation of testing data via MLPNN ensemble modelling.

### 4.3. Random Forest Model Outcomes

**Figure 10:**
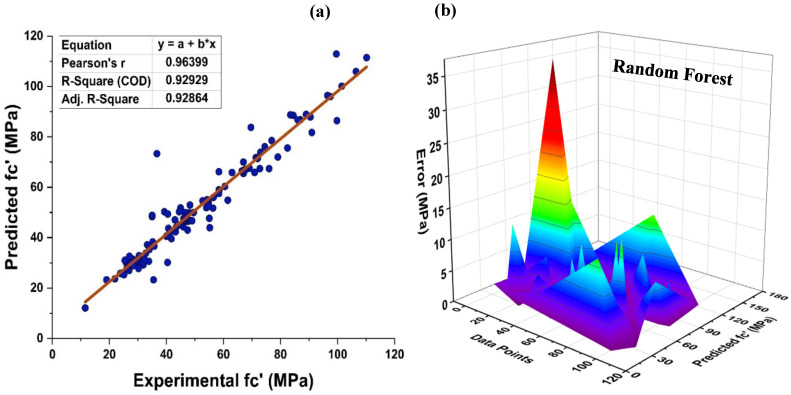
(**a**) RF modified learner regression model; (**b**) RF modified learner regression model error distribution.

Within the framework of the ensemble ML approach, RF represents a hybrid type of bagging and random feature selection, which is a technique for the production of prediction models that is both efficient and easy to use. Figure 10 depicts the prediction accuracy of the RF method for MK concrete. As it is an ensemble model, it exhibits a stubborn R^2^ = 0.929 correlation with the target values. In addition, the RF model’s prediction may also be tested using an error distribution with an average error of 3.52 MPa. In addition, 90.99 percent of the results indicate that the error falls under 10 MPa, demonstrating the precision of the non-linear estimation of the normal concrete’s strength as shown in Figure 10b.

### 4.4. K-Fold Results

**Figure 11:**
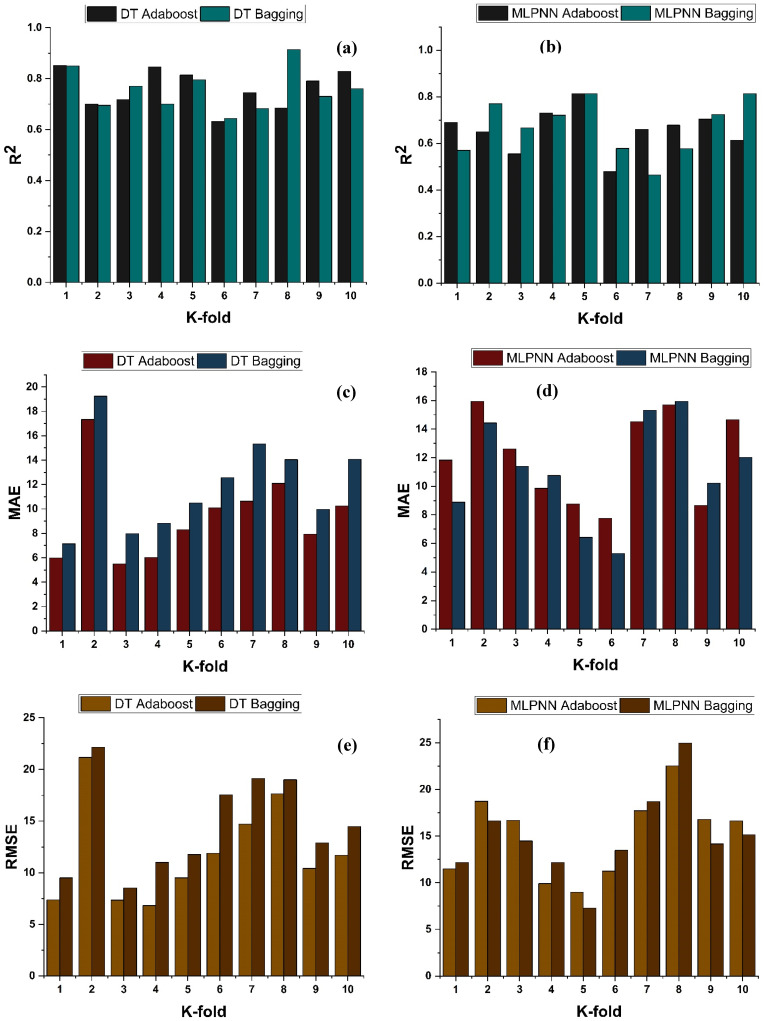

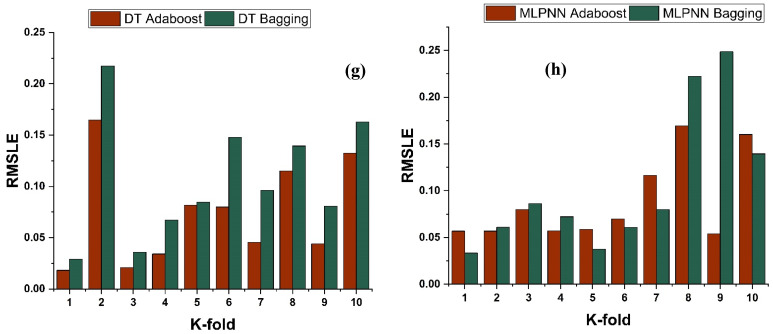
fc’ models (**a**,**b**) indicate R^2^ models’ result validated with K fold; (**c**,**d**) indicate MAE models’ result validated with K fold; (**e**,**f**) indicate RMSE models’ result validated with K fold; (**g**,**h**) indicate RMSE models’ result validated with K fold.

**Figure 12:**
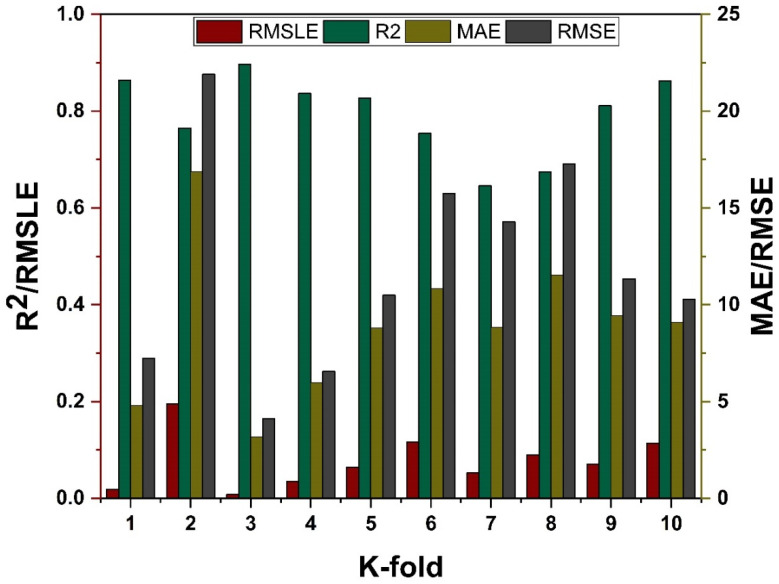
RF models cross validation with different statistical parameters.

The model’s required accuracy is essential to its assessment. To verify the accuracy of prediction models, this validation is necessary. The K fold validation test is employed to validate the correctness of data using data shuffles. Randomly sampling the training data set introduces bias, hence this strategy is used to reduce it. This technique divides the samples evenly into 10 subgroups of the experimental data. One of the 10 subsets is utilised for validation, while the other nine are employed to shape up the strong learner. The procedure is done 10 times and then averaged. In general, it is commonly accepted that the 10-fold cross validation approach accurately reflects the model’s generalisation and dependability [79]. The validation test of all the ensemble models is presented in Figure 11 and Figure 12. All models exhibit a moderate to high correlational link. In addition, the outcomes of cross-validation may be evaluated based on various errors, such as R^2^, MAE, RMSE, and RMSLE, as shown in Figure 11 for DT and MLPNN and Figure 12 for RF. It displays the validation representation in each 10-fold. Although variations were noticed, it retained a high degree of precision, as seen in Figure 11 and Figure 12. For example, the lowest and highest R^2^ values for all models are between 0.46 and 0.65 and 0.81 and 0.91, respectively. As demonstrated in Figure 11c–h for DT and MLPNN, MAE, RMSE, and RMSLE are also used to evaluate the accuracy of models with respect to cross-validation. Figure 11c depicts the average MAE value for DT with ensemble bagging and ensemble boosting using 10-fold validation as 11.97 MPa and 9.0 MPa, respectively. Figure 11e reveals that the RMSE offers an average error of about 14.6 MPa and 11.84 MPa for ensemble bagging and ensemble boosting using AdaBoost, respectively. Figure 11g displays RMSLE average errors of 0.106 MPa and 0.07 MPa for DT bagging and boosting, respectively. Figure 11d–f shows that the average MAE, RMSE, and RMSLE for the MLPNN bagging model are 11.06 MPa, 14.92 MPa, and 0.1 MPa, respectively. For the k fold validation of the MLPNN AdaBoost model, values of 12.03 MPa, 15.07 MPa, and 0.08 MPa were found. This demonstrates the precision of models using K-fold cross validation. Figure 12 demonstrates strong association for modified learner model with decreased error for MAE, RMSE, and RMSLE, with average errors of 8.94 MPa, 11.02 MPa, and 0.07 MPa, respectively.

### 4.5. Model Evaluation and Discussion Based on Statistical Metrics

**Figure 13:**
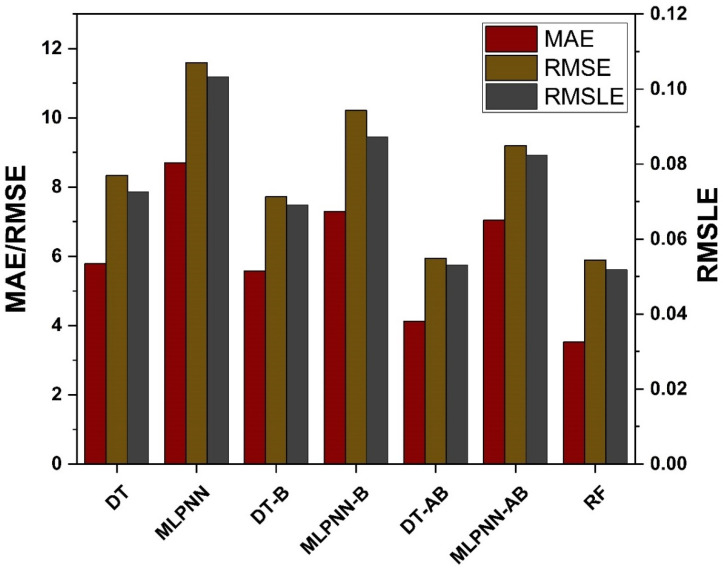
Statistical analysis of fc’ models.

Comparing the ensemble approaches to the individual ML methods helped show the ensemble algorithm’s potential in comparison to them as depicted in Figure 13. This process is similar to that used for ensemble models, such as starting with a set of values and then using a grid search to find the optimal values. Table 5 shows the target and validated values for each metric. The ensemble ML models outcome have a linear trend, and their projections are more like the ones that were tested, according to this study. Using DT, and MLPNN, is a kind of individual learning, but using ML techniques such as bagging and boosting is a form of ensemble learning. High performance weak learners would gain weight, though weak learners with poor performance will lose weight, since ensemble learning is usually known to include several weak learners produced by individual ML algorithms. Because of this, it is able to provide accurate projections. MAE, RMSE, RMSLE, and R^2^ are used to evaluate individual and ensemble learners. An ensemble of learners using bagging and boosting has a lower rate of error than an individual learner. A smaller error margin exists between forecasts and outcomes when using ensemble models rather than individual models alone.

### 4.6. SHAP Analysis

**Figure 14:**
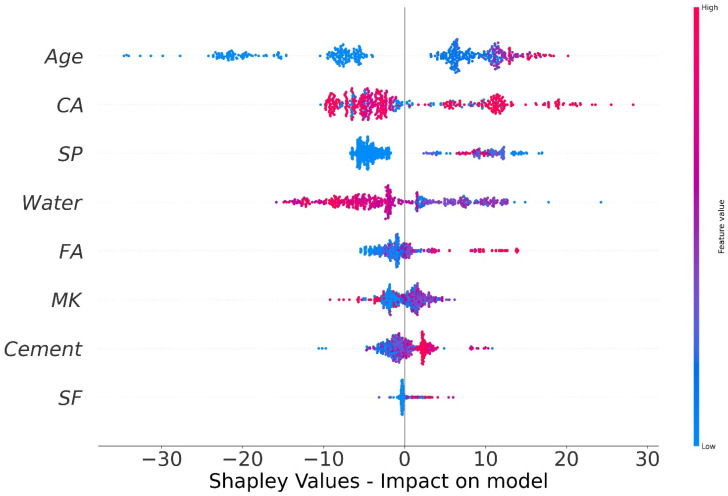
SHAP plot.

**Figure 15:**
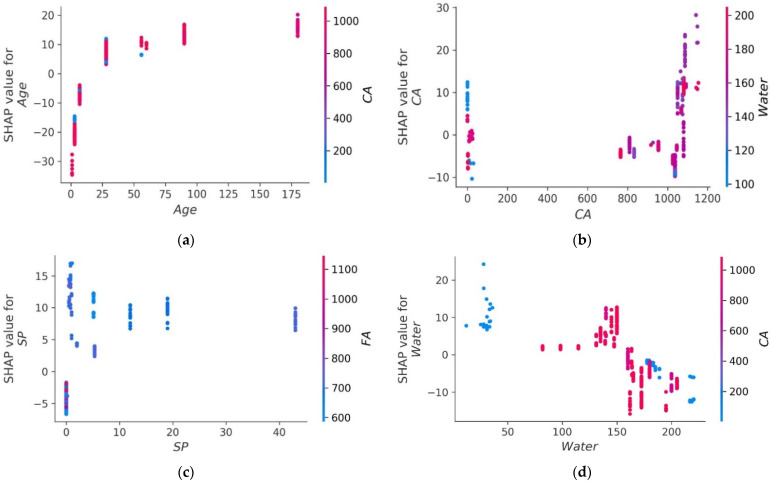

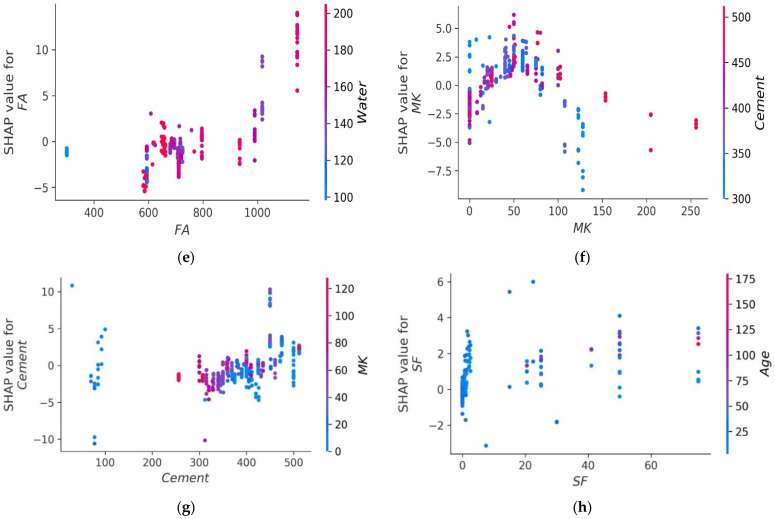
SHAP interaction plot of parameters: (**a**) age; (**b**) coarse aggregate; (**c**) superplasticizer; (**d**) water; (**e**) fine aggregate; (**f**) metakaolin; (**g**) cement; (**h**) silica fume.

The values of all of the features that were taken into consideration for the MK concrete fc’ prediction are outlined in the shape of a violin, as illustrated in Figure 14. The Shapley value measures the mean marginal influence that can be attributed to each parameter value over all viable permutations of the parameters. The attributes that have substantial absolute Shapley values are regarded to have a considerable influence. In order to get the global feature effects, the absolute Shapley values for each feature throughout the data were averaged and ranked in decreasing significance as shown in Figure 14. Every single datapoint on the plot indicates a Shapley value for distinct characteristics and occurrences. The location on the x axis and the y axis is defined by the Shapley value and the feature significance, respectively. Elevated places on the y axis represent higher effect of the characteristics on the MK concrete fc’ prediction and the colour scale reflects the feature relevance from low to high. Each dot in Figure 14 signifies an individual point from the dataset. The location of points along the x axis represents the effect of each parameter value on the fc’ prediction. When numerous dots fall in the same location along the x-axis, the dots are stacked to illustrate the density. Age is the most influential parameter followed by coarse aggregate, superplasticizer, water, and other input parameters. Silica fume has the least impact on fc’ prediction of MK concrete as illustrated in Figure 14. Higher SHAP value imply that the model forecasts higher fc’ value, and vice versa. For example, high value of age (red) correlate with increased SHAP value, which suggest high fc’ value. Moreover, each input parameter has positive or negative impact up to a certain limit. In Figure 14, red colour shows high impact (negative or positive) while the blue colour depicts low impact of the input feature on the predicted outcome. SHAP value at the right (greater than 0) on the x-axis shows positive impact of respective input on the fc’. For instance, in the case of input parameters like age and content of coarse aggregate, the positive effect of these factors on MK fc’ can be noted from the right axis of the graph. Coarse aggregate content depicts a constructive impact till optimum content, whereas above this content, the adverse effect is shown on the left side (less than 0) on the x-axis. Super-plasticizer is also key variable for predicting the fc’ of MK concrete. The effect of water on the output fc’ of MK concrete is negative and increasing the water content will reduce the fc’. MK and cement show the same trend. However, SF and fine aggregate tend to have a high positive impact and a low negative impact on the fc’ prediction of MK concrete. SHAP feature dependency graphs were deployed that are coloured by another interacting feature to highlight how the features interact and effect the fc’ of MK concrete. This gives greater information than standard partial dependency charts. The SHAP interaction plot each considered feature is shown in Figure 15. As can be observed from Figure 15a, the dependence and interaction show that high fc’ values for MK concrete can be achieved when for 50 ≤ age ≤ 100 days when CA ≥ 700 kg/m^3^. Higher fc’ values for age ≤ 50 days can be achieved for 50 ≤ CA ≤ 700 kg/m^3^. Figure 15b,d,e show that 120–200 kg/m^3^ of water is required for CA in for different content of CA and FA to achieve higher values of fc’. Moreover, Figure 15f,g illustrate the relation between two important constituents of MK concrete: cement and metakaolin. Higher fc’ for MK concrete can be achieved for MK in the range of 20–100 kg/m^3^ for concrete having density of 250–450 kg/m^3^. Additionally, Figure 15h reveals that large quantity of silica fume can be used if early fc’ of MK concrete is desired.

## 5. Conclusions

The primary aim of this study was to assess the accuracy level achieved by various ML approaches to predict MK concrete fc’. Datasets from the literature containing 551 data points were used to train and test the models. The eight most influential constituents of MK concrete including cement, metakaolin, coarse and aggregate, water, silica fume, superplasticizer, and age were considered as input parameters. Individual and ensemble learning models for predicting the fc’ of MK concrete were investigated in this study using DT, MLPNN, and RF. Interaction of input parameters and effect of input parameters of fc’ were studied using SHAP dependency feature graphs. The results of the investigation led the authors to the following conclusions:

1. Bagging and AdaBoost models outperform the individual models. As compared to the standalone DT model, the ensemble DT model with boosting and RF demonstrates a 7% improvement. Both techniques have a significant correlation with R^2^ equal to 0.92. Similarly, an improvement of 14 %, 6%, and 29% was observed in MLPNN AdaBoost, MLPNN bagging, and RF model, respectively, when compared with individual DT model;
2. Statistical measures using MAE, RMSE, RMSLE, and R^2^ were also performed. Ensemble learner DT bagging and boosting depicts a smaller error of about 4%, and 29% for MAE, 8% and 29% for RMSE, 5% and 27% for RMSLE, respectively, when compared to the individual DT model. Similarly, enhancements of 16% and 19% in MAE, 12% and 21% in RMSE, and 16% and 20% in RMSLE were observed for MLPNN bagging and AdaBoost models, respectively, when compared to the individual base learner DT model;
3. RF shows improvements of 60%, 49%, and 50% in MAE, RMSE, and RMSLE when compared to the MLPNN individual model. Similarly, improvements of 39%, 29%, and 29% for the RF model, in MAE, RMSE and RMSLE, were observed in comparison to DT individual model;
4. The validity of models using R2, MAE, RMSE, and RMSLE were tested using k-fold cross-validation. Fewer inaccuracies with strong correlations were examined;
5. The DT AdaBoost model and the modified bagging model are the best techniques for forecasting MK concrete fc’ among all of the ML approaches;
6. Age has the greatest impact on calculating MK concrete fc’, followed by coarse aggregate and superplasticizer, according to the SHAP assessment. However, silica fume has the least impact on the fc’ of MK concrete. SHAP dependency feature graphs can illustrate the relationship between input parameters for various ranges;
7. Sensitivity analyses depicted that FA contributed moderately to the development of the f_c_’ models and f_sts_ models. Moreover, cement, SF, CA, and age played vital roles in the development of f_c_’ models. Tensile strength models showed to be affected least by water and CA;

These ML algorithms can accurately predict the mechanical characteristics of concrete. These models can be utilized to predict the mechanical characteristics of similar databases containing metakaolin with high accuracy. Moreover, SHAP analysis provides an insight to readers regarding the input parameters contribution towards the outcome, and inter-dependency of the input parameters. This will enable the readers to carefully select the input variables for modelling the behaviour of metakaolin concrete. Additionally, ML algorithms employed in this study may provide a sustainable way for the mix design of MK concrete. Traditionally, this process demands lengthy trials in laboratories and a significant number of raw materials in addition to a great deal of manpower.

## 6. Limitations and Directions for Future Work

Despite the fact that the efforts made in this research has significant limitations, it may still be regarded a data mining-based research. Completeness of dataset is essential for the efficacy of models’ prediction. The range of datasets used for this study was restricted to 551 data points. In addition, the corrosive and flexural concrete behaviour at extreme temperatures was not considered in this work. Indeed, good database management and testing are essential from a technical standpoint. To simulate high-strength concrete, this research included an extensive variety of data with eight variables. Further, it is suggested that a new dataset of concrete at increased temperatures that encompasses numerous environmental factors such as temperature, durability, and corrosion be investigated. Experimental testing data for testing of models are recommended for more accuracy. Given that concrete plays such an important role in the ecosystem, its effects under various situations should be investigated utilising various deep machine learning methods.

## Figures and Tables

**Table 1:** Metakaolin concrete compressive strength model input and output variable ranges.

Parameters	Acronym	Min	Max
Input Parameters			
Cement	C	30	512
Fine aggregate	FA	300	1146
Coarse aggregate	CA	0	1154
Water	W	12	220.30
Silica fume	SF	0	75
Metakaolin	MK	0	256
Superplasticizer	SP	0	43
Age	Age	1	180
Output variable			
Compressive strength	fc’	9.84	131.30

**Table 2:** Statistical description of Metakaolin concrete variables.

Parameters	Cement	Fine Aggregate	Coarse Aggregate	Water	Silica Fume	Metakaolin	Superplasticizer	Age (Days)
Statistical description								
Mean	396.64	737.24	853.09	161.71	4.05	45.48	2.90	37.29
Std error	3.72	7.79	15.47	1.53	0.55	2.16	0.33	1.93
Median	400	711	1037	163.40	0	40	0	28
Std. dev	87.28	182.93	363.13	36.00	13.02	50.59	7.73	45.34
Variance	7617.40	33,463.99	131,866.26	1295.65	169.49	2559.62	59.80	2056
Kurtosis	3.57	0.37	1.42	4.18	13.68	4.43	16.38	3.13
Skewness	−1.34	0.52	−1.75	−1.58	3.68	1.85	3.92	1.90
Range	482.00	846	1154	208.30	75	256	43	179
Min	30	300	0	12	0	0	0	1
Max	512	1146	1154	220.30	75	256	43	180
Sum	218,548.70	406,217	470,050.80	89,099.50	2232.15	25,060.77	1597.30	20549
Count	551	551	551	551	551	551	551	551
Training dataset								
Mean	395.18	738.49	841.41	161.06	4.09	45.50	2.66	37.85
Std error	4.19	8.86	17.83	1.74	0.63	2.39	0.35	2.20
Median	400	711	1037	163.40	0	40	0	28
Std. dev	87.97	185.84	374.05	36.54	13.24	50.03	7.38	46.20
Variance	7738.04	34,538.02	139,915.86	1334.83	175.26	2503	54.44	2134.15
Kurtosis	3.65	0.33	1.04	4.08	13.61	4.56	18.52	2.92
Skewness	−1.38	0.52	−1.66	−1.57	3.69	1.86	4.13	1.86
Range	482	846	1154	208.30	75	256	43	179
Min	30	300	0	12	0	0	0	1
Max	512	1146	1154	220.30	75	256	43	180
Sum	173,881.30	324,936.00	370,219.70	70,868.40	1798.72	20019.51	1171.52	16656
Count	440	440	440	440	440	440	440	440
Testing Dataset								
Mean	402.41	732.26	899.38	164.24	3.90	45.42	3.84	35.07
Std error	8.03	16.29	29.75	3.21	1.15	5.03	0.85	3.98
Median	400	708	1037	163.40	0	40	0	28
Std. dev	84.64	171.61	313.42	33.81	12.16	53	8.98	41.92
Variance	7163.11	29,450.55	98,231.83	1142.93	147.97	2808.83	80.63	1756.94
Kurtosis	3.30	0.59	3.85	4.79	14.25	4.19	11.32	4.29
Skewness	−1.16	0.54	−2.25	−1.62	3.64	1.85	3.31	2.06
Range	434.50	846	1149	192.40	75	256	43	179
Min	77.50	300	0	27.90	0	0	0	1
Max	512	1146	1149	220.30	75	256	43	180
Sum	44,667.40	81,281.00	99,831.10	18,231.09	433.43	5041.26	425.78	3893
Count	111	111	111	111	111	111	111	111

**Table 3:** DT model statistical evaluation of errors.

Statistical Analysis	DT	DT-Bagging	DT-AdaBoost
Average	5.79	7.29	7.05
Minimum	0.08	0.11	0.07
Maximum	35.3	34.74	31.31
No. of data points below 10 MPa	92	94	103
No. of data points between 10 and 20 MPa	15	14	07
No. of data points between 20 and 30 MPa	02	02	00
No. of data points between 30 and 40 MPa	02	01	01
No. of data points testing points	111	111	111
Average below 10 MPa	82.88	84.68	92.79
Average in range of 10 to 20 MPa	13.51	12.61	6.31
Average in range of 20 to 30 MPa	1.80	3.60	00
Average in range of 30 to 40 MPa	1.80	2.70	0.90

**Table 4:** MLPNN model statistical evaluation of errors.

Statistical Analysis	MLPNN	MLPNN-Bagging	MLPNN-AdaBoost
Average	8.70	7.29	7.05
Minimum	0.04	0.11	0.07
Maximum	35.15	34.74	31.31
No. of data points below 10 MPa	81	86	83
No. of data points between 10 and 20 MPa	20	18	23
No. of data points between 20 and 30 MPa	07	04	04
No. of data points between 30 and 40 MPa	03	03	01
No. of data points between 10 and 20 MPa	111	111	111
Average below 10 MPa	72.97	77.48	74.77
Average in range of 10 to 20 MPa	18.02	16.22	20.72
Average in range of 20 to 30 MPa	6.31	3.60	3.60
Average in range of 30 to 40 MPa	2.70	2.70	0.90

**Table 5:** Model’s statistical errors.

Approach Employed	ML Methods	MAE	RMSE	RMSLE	R^2^
Individual Learner	DT	5.79072	8.34472	0.07261	0.868
	MLPNN	8.70159	11.59452	0.10325	0.724
Ensemble Learner Bagging	DT	5.57845	7.72089	0.06911	0.879
	MLPNN	7.29168	10.21239	0.08721	0.767
Ensemble Learner Boosting	DT	4.12636	5.93813	0.05303	0.924
	MLPNN	7.04574	9.20414	0.08233	0.825
Modified Ensemble	Random Forest	3.52232	5.89161	0.05179	0.929

## Data Availability

All data is available in the paper.
